# Influence of dental treatment in place on quality of life in oral cancer patients undergoing chemoradiotherapy

**DOI:** 10.4317/medoral.22353

**Published:** 2018-06-21

**Authors:** Jesús Núñez-Aguilar, Luis-Guillermo Oliveros-López, Ana Fernández-Olavarría, Daniel Torres-Lagares, Mª Angeles Serrera-Figallo, Aida Gutiérrez-Corrales, Jose-Luis Gutiérrez-Pérez

**Affiliations:** 1 PhD, DDS, MSc. University of Seville; 2 DDS, MSc. Oral Surgery Residents. University of Seville; 3 PhD, DDS, MSc. Professor of Oral Surgery. Chairman of Oral Surgery. Department of Stomatology. University of Seville; 4 PhD, DDS, MSc. School of Dentistry. University of Seville; 5 PhD, DMD, Professor of Oral Surgery. Chairman of Oral Surgery. Department of Stomatology. University of Seville

## Abstract

**Background:**

This study aims to assess, in the population of patients with oral cancer treatment, the influence on the quality of life of two protocols of dental treatment: not ruled hospital treatment versus ruled hospital treatment.

**Material and Methods:**

A quasi-experimental approach justified on ethical grounds was used. A total of 41 patients were included in the control group (not ruled treatment outpatient health center) and 40 in the experimental group (ruled hospital treatment). A total of 14 questions to both groups were conducted in three stages: before starting cancer treatment, during treatment and after treatment. the proportions of positive responses in groups and different times were compared using the chi-square test.

**Results:**

Based on similar situations during cancer treatment were identified as six issues favorable to the experimental group difference. This number rose to nine after finishing oncological treatment.

**Conclusions:**

From our data we can confirm that planned dental treatment performed during the oral cancer treatment produces an improvement in the quality of life in patients with oral cancer.

** Key words:**Oral cancer, dental treatment, quality of life, oncology, dentistry.

## Introduction

The term cancer is used to name a group of diseases with abnormal and uncontrolled proliferation of cells that invade close and distant organs. The incidence rates of oral and oropharyngeal cavity cancer have increased in recent years. (1) Head and neck carcinomas represent 5% of all cancers in men and 2% in women; the most frequent location of malignant primary tumors of the head and neck is the oral cavity (1,2). In the European Union, the annual incidence is 48.9 cases per 100,000 individuals and the mortality is 30.8 cases per 100,000 inhabitants. The annual prediction worldwide is about 500,000 new cases. Oropharyngeal carcinomas represent 40% of head and neck carcinomas (1).

Smoking, alcohol and poor oral hygiene are considered the main etiological predisposing factors to the development of this malignancy (3). Other predisposing etiological factors, such as infection by the human papillomavirus and the presence of chronic oral inflammation, have also been described. The latter two conditions play an important role in patients who have never been smokers or drinkers (4,5).

The tumor localization and the treatment method, along with the stage of the disease, play an essential role not only in the prognosis of head and neck cancers, but also in the incidence and intensity of side effects (6,7). The improvement in the different treatments of head and neck cancers have decreased the mortality of these patients but, on the other hand, the patient must live with the different sequelae of this disease, which affect their quality of life (8,9).

The aftermath of the surgical resection of the tumor or the different sessions of radiotherapy and chemotherapy affect speech, swallowing, chewing and salivation, which in turn result in the nutritional deficit of the patient. In addition, there will be aesthetic and psychological consequences (10,11). Some of the side effects inherent to cancer treatment, such as oral mucositis, xerostomia and caries, which directly affect the quality of life of patients and can be controlled in the dental office (12).

There are several studies within the scientific literature in which the beneficial effects of dental therapies in the oral health of patients diagnosed with oral cancer and subsequent positive impact on their quality of life (10,12-16) are observed, but there is not enough scientific information that described the quality of life of patients throughout the cancer therapy. For this reason, the aim of our study is to assess the influence of regulated dental treatment in the quality of life of patients with oral cancer during cancer therapy (before, during, after).

## Material and Methods

We conducted a quasi-experimental study at the Oral and Maxillofacial Surgery Service of Virgen del Rocio Hospital in the city of Seville. In mid 2005, the possibility of incorporating some degree of dental treatment for cancer patients in the same hospital was raised (along the lines of Kielbassa *et al.*) (17).

We received the proposal by the hospital management, but we needed time to implement the necessary resources; therefore, we began this study by including patients with oral cancer (Squamous Cell Carcinoma) within the control group. A total of 41 patients were received from September 2005 to September 2006. Once we implemented the necessary resources to provide dental treatment, the 40 patients who comprised the experimental group began to receive treatment between October 2006 and October 2007.

All were treated at Integral Consultation of Oropharyngeal Tumors of Virgen del Rocio Hospital in Seville; after evaluating their tumor process, were they were scheduled to receive cancer treatment. At that time, the patients’ state of dental health, habits and oral problems were assessed. The patients in the control group were informed and advised of the care they should have during the chemoradiotherapy treatment. This approach was established and monitored at primary care level centers (Table 1). The experimental group patients were under regulated dental treatment, following the guidelines of Kielbassa *et al.* (26) ( Table 1), which was integrated with the treatment of their cancer. This protocol was performed in the facilities of the hospital.

**Table 1:**
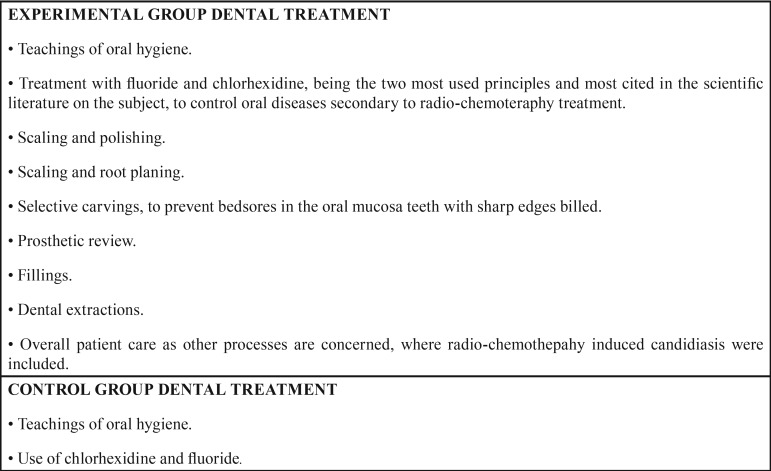
List of treatments applied in the hospital to the patients in each group.

The study protocol was approved by the Ethics Committee of the Virgen del Rocio Hospital (Code USE-DENT-2005). All patients read and signed the informed consent to participate in the study and the guidelines outlined in the Declaration of Helsinki for human experimentation were followed.

The inclusion criteria applied in this study were as follows: Patients diagnosed with oropharyngeal cancer (squamous cell carcinoma) belonging to the Virgen del Rocio Hospital in Seville, in need of combined cancer treatment, chemoradiotherapy, that have not undergone surgery, were not edentulous and demonstrated a Karnosfsky index equal to or greater than 50%.

The exclusion criteria applied in this study were as follows: Patients who voluntarily refused the proposal made by the specialists of our service and who decided to make another treatment alternative to oncologists indicated primarily for treatment; Patients who were referred to another hospital; Patients who chose not to be treated for their disease; Patients who willingly left cancer treatment; Patients who refused to submit to any part of the study or refused to consent to the scientific use of their data by not signing or citing a breach of informed consent; patients who died during the study.

To assess the influence of different dental treatments that were implemented in the quality of life of patients, a questionnaire consisting of a battery of short-answer questions (yes or no) was performed among patients in both groups. The questionnaire was designed to provide an idea of the perception of the state of buco-dental health and the patients’ experience with oral problems experienced during treatment. In addition, four questions on the general state of the patient and their mood were introduced in an effort to drive the individual into a more psychic than somatic dynamic.

In each group, the survey was conducted before (one month before the chemoradiotherapy), during (when the patients had completed 60% of the chemoradiotherapy) and after cancer treatment (twelve months after starting the study) ( Table 2).

**Table 2:**
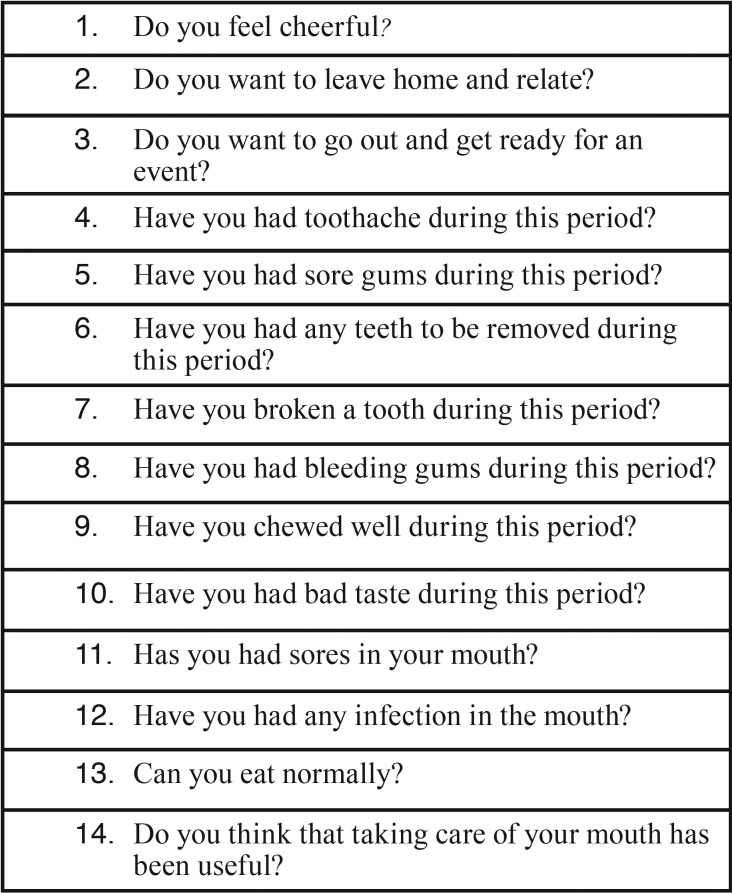
Questions asked of the patients in the study. Positive responses to the interest of the study were assessed. For example, in question 13, the answer Yes is considered positive, whereas, for example, in question 8, the answer was not considered positive.

The answers to these questionnaires were codified in a data file by using SPPS v.11 (IBM, USA) for statistical analysis. The descriptive study based on the percentage of responses in a positive sense (which does not always coincide with “Yes”). To identify differences that could be significant between the two groups, the chi square test was applied. The Student t test method has been used to obtain a comparative mean of positive responses in each group and at each time of the study. The authors applied the Kolmogorov-Smirnoc test to ensure the normal distribution of the data.

## Results

**Figure 1:**
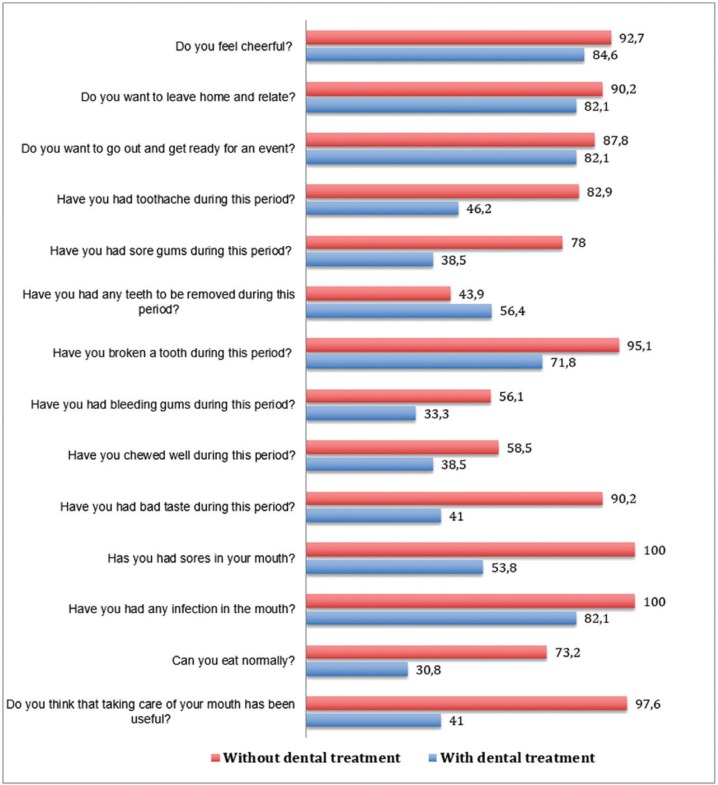
Survey about the quality of life before cancer treatment according to group (percentage (%) of positive responses).

We begin this section by pointing out the comparability between the quality of life of groups of patients before treatment of chemoradiotherapy (Fig. 1). The questionnaires collected before starting treatment with chemoradiotherapy showed no major differences between the two groups. Although there were significant differences in seven questions (8), in four of them, the differences were in favor of the control group.

**Figure 2:**
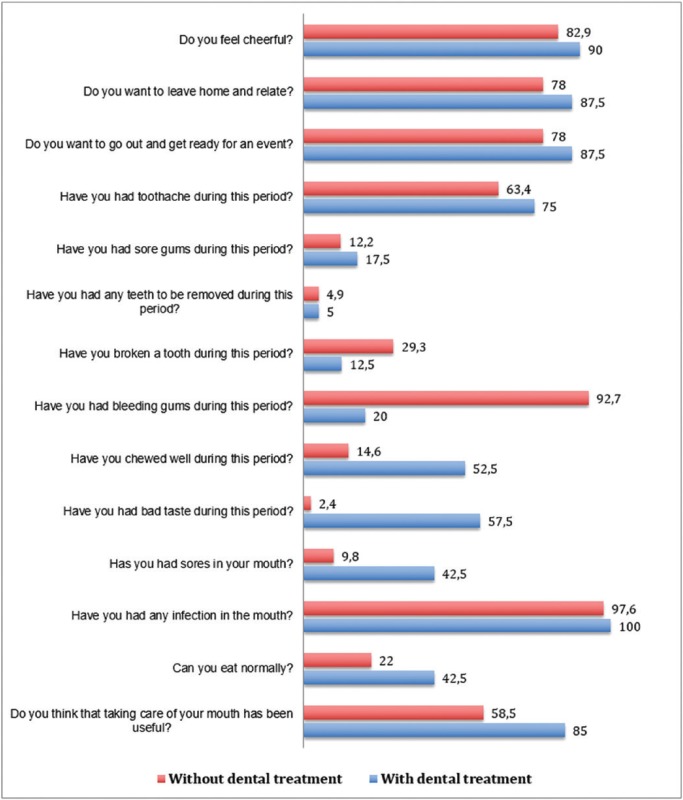
Survey about the quality of life during cancer treatment according to group (percentage (%) of positive responses).

Regarding the influence of dental treatment on the quality of life of patients during chemoradiotherapy (Fig. 2), once established cancer treatment (when the patients had completed 60% of the chemoradiotherapy) had begun, the questionnaires revealed a positive trend toward the regulated dental treatment group, although not all questions represented statistically significant differences. Only 6 questions were identified as significant differences, all in favor of the experimental group.

The results of the questions with significant differences in each group are shown: Have you had sore gums during this period? In the experimental group, 20 patients (47.78%) indicated that they had not had sore gums during this phase of the chemoradiotherapy. In the control group, 35 patients (87.5%) reported sore gums at this stage; we found significant difference between the groups (*p* <0.001). Another question was have you had bleeding gums during this period? In the experimental group, 7 patients (17.07%) told us they had not had bleeding gums during chemoradiotherapy. In the control group, 35 patients (87.5%) reported having bleeding during this phase (*p* <0.001). In response to the question, Have you chewed well during this period? In the experimental group, 21 patients (51.21%) said that they were able to chew well at this stage. In the control group, 6 patients (14.63%) said that they could chew well, so we found no significant difference between groups (*p* <0.001). In response to Have you had a bad taste during this period? In the experimental group, 18 patients (43.9%) reported having a bad taste. In the control group, 39 patients (97.5%) reported having no bad taste at this stage of chemoradiotherapy (*p* <0.001). In response to Have you had mouth ulcers during this period? In the experimental group, 4 patients (9.75%) had ulcers during chemoradiotherapy. In the control group, 35 patients (87.5%) indicated that they had mouth ulcers, finding significant differences between the two groups (*p* <0.001). Another question was Do you think that taking care of your mouth has served your health well? In the experimental group, 34 patients (82.92%) said that it helped during chemoradiotherapy. In the control group, 24 patients (58.53%) indicated that taking care of their mouth served them well during this phase (*p* = 0.01).

**Figure 3:**
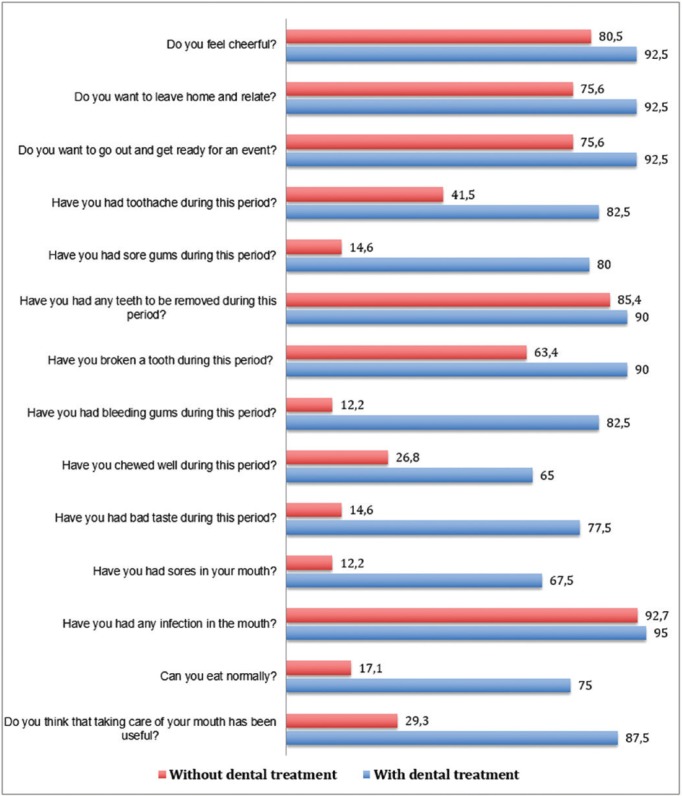
Survey about the quality of life after cancer treatment according to group (percentage (%) of positive responses).

Finally, we assessed the influence of dental treatment on the quality of life of patients after chemoradiotherapy (Fig. 3). After cancer treatment, twelve months after starting the study, the number of questions with significant differences between both groups increased to nine, all in favor of the experimental group.

The results of the questions with significant differences in each group are shown: Do you want to leave home and relate? In the experimental group, 38 patients (92.68%) said that they want to leave home, in the control group 31 patients (75.60%) indicated that they want to leave home (*p* = 0.03). Another question was, Do you want to go out and get ready for an event? The data in this section are similar to those of the previous question; in the experimental group, 38 patients responded to be ready after chemoradiotherapy and, in the control group, 31 patients expressed the desire to go out (*p* = 0.03). Further, when asked the question, Have you had a toothache during this period?, 7 patients (17.07%) in the experimental group stated that they had a toothache after chemoradiotherapy. In the control group, 23 patients (57.5%) had toothaches during this period—we found a significant difference between the two groups during this phase (*p* <0.001). With regard to the question, Have you had sore gums during this period?, 8 patients (19.51%) in the experimental group affirmed that they had sore gums during this period after chemoradiotherapy. In the control group 23 patients (57.5%) had sore gums after chemoradiotherapy (*p* <0.001). Lastly, we asked the patients, Have you had bleeding gums during this period? In the experimental group, we found a total of 7 patients (17.07%), whose gums bled after chemoradiotherapy. In contrast, in the control group, a total of 36 patients (87.80%) stated that their gums bled after chemoradiotherapy sessions, thus revealing statistically significant differences between groups (*p* <0.001).

Other questions with significant differences between the two groups were: Have you chewed well during this period? In the experimental group, 27 patients (65.85%) indicated that they were able to chew well after chemoradiotherapy. In the control group, 11 patients (26.82%) said they chewed well during this phase (*p* <0.001). With regard to the question, Have you had bad taste in your mouth?, 9 patients (21.95%) in the experimental group responded that they had bad taste after chemoradiotherapy. In comparison, 34 patients (85%) in the control group said they had no bad taste in this period (*p* < 0.001). When asked, Can you eat normally?, 31 patients (75.60%) in the experimental group said that they usually ate after chemoradiotherapy. In the control group, 7 patients (17.07%) indicated that normally ate after anti-tumor therapy (*p* <0.001). In response to the question, Do you think that taking care of your mouth has been useful?, 36 patients (87.80%) in the experimental group answered that it has been useful after chemoradiotherapy. In the control group, 12 patients (29.26%) answered that mouth care has been beneficial (*p* <0.001).

If we value the evolution of the positive answers in each group, then we clearly see how this variable goes from 11.46 ± 1.3 to 6.41 ± 1.1 in the control group throughout the cancer treatment while, in the experimental group, it declined from 7.63 ± 1.4 to 11.7 ± 1.8. These differences at the beginning and the end of the study were statistically significant (*p* < 0.05) between the two groups, as well as in the same group between the start and the end point of the study.

## Discussion

Dentists play a major role in the early detection of oral cancer and the maintenance of oral health for patients during all stages of this disease, but their role in addressing this disease should not stop here (10,12-16,18,19).

Since 1994, publications have recommended the establishment of previous dental therapies for cancer treatment in patients with head and neck cancer. Lockhart and colleagues recommend a thorough treatment of the patient by the dentist to prevent complications during and after radiotherapy, thus providing a better quality of life (20).

Currently, the consequences and side effects of cancer therapies in patients with oral cancer and the effect of these therapies in their quality of life are well known (8,9,12). Oral mucositis is one of the major side effects and is the most important of the chemotherapy and radiotherapy. It hinders patients’ food intake, causes alterations in taste and limits speech and chewing ability while causing pain, thus decreasing the quality of life of patients (21-23). A 2008 report revealed that 80% of patients suffering from head and neck carcinoma suffer from severe oral mucositis (11).

In 2013, McGuire *et al.* (22) conducted a systematic review regarding care of the oral cavity in the management of oral mucositis in cancer patients. They concluded that there is scientific evidence on the use of oral hygiene protocols among patients receiving radiotherapy and chemotherapy for the prevention of oral mucositis. They also found that the use of chlorhexidine does not prevent mucosal inflammation after cancer therapies. Similar results were obtained by Diaz Sanchez *et al.* (23) in 2015. In their study on the use of bioadhesive chlorhexidine gel in the prevention and treatment of mucositis induced by chemotherapy in the head and neck, they did not observe the relief of pain and discomfort among patients.

In 2015, Barrios *et al.* (10) conducted a study of 142 patients on the relationship between oral health and quality of life of patients suffering from oral cancer surgery, radiotherapy and/or chemotherapy. They found that oral health and quality of life of patients decreased at least six months after cancer treatment. They also recommended the implementation of rehabilitation programs to benefit and improve the quality of life of patients. These results differ from those obtained in our study, although we believe that this is due to the inclusion criteria as they treated patients undergoing surgical therapy of tumor resections, leading to more complicated patient management that affected their oral health and quality of life.

Some studies have shown that the surgical treatment of oral cancer increases survival of oral cancer patients, but the physical changes caused difficulty with opening the mouth, chewing and speaking, as well as obtaining good nutrition and social relations, among other aspects, thus directly affecting the patients’ quality of life (6,24).

In 2016, Peisker and colleagues (25) conducted a long-term study on the quality of life of patients with oral squamous cell carcinoma undergoing cancer surgery with the vascularized reconstruction of a free flap. They concluded that the surgery was successful, but the patient’s quality of life was diminished. So, after cancer treatment, they recommended establishing psychological therapies, nutritional treatment and rehabilitation as part of the multidisciplinary care for the patients.

Quality of life and its measurement values are becoming more important every day in oncological therapies. Therefore, several recent studies have been published in the scientific literature (25). The specific system to objectively quantify the quality of life of patients has been and remains difficult, yet there are several valid and useful methods in the field of oncology. We found the basic questionnaires of the European Organization for Research and Treatment of Cancer Quality of Life version 3.0 (EORTC QLQ-C30), specifically the head and neck modules (EORTC QLQ-HN35 and (FACT-HN), the generic questionnaire Short Form-36 (SF-36) and others (26). The difference between these questionnaires and the one we used in our study is very clear and must be highlighted. The questionnaires described different measure scales (physical, role, cognitive state, state emotional, social, symptoms, sensory, sexuality), yet our questionnaire is based on oral health data, subjective information and prior psychological data, during and after cancer therapy, that allow us to observe a significant improvement in the quality of life of patients in this study.

Reviewing the results obtained in the control group, we observed that there are statistically significant differences in improving the quality of life of these patients.

It should be highlighted that no dental treatment was performed in the control group; patient follow-up and monitoring occurred via the corresponding administration of toothpaste, mouthwashes and oral hygiene education and then derive to the reference ambulatori center. Although treatment in this group was limited to advice and instruction in mouth care, its beneficial effect on the patient has been demonstrated. Therefore, our study should not only be assessed on the dental treatment, but also on the organization of such treatments around the cancer therapy.

In our study, before chemoradiotherapy treatment, research groups had similar situations. During chemoradiotherapy, the situation changed dramatically. Patients in the experimental group had fewer gum symptoms (*p* <0.001); there was a decrease in gums bleeding (*p* <0.001) and less proliferation of oral ulcers. Patients chewed better during cancer therapy (*p* <0.001), which could result in better feeding and prevention of possible malnutrition.

After chemoradiotherapy, we found several results with statistically significant differences in both groups to consider. Patients in the experimental group had fewer dental symptoms and less gum pain (*p* <0.001). Patients in the control group experienced more bleeding gums (*p* <0.001), while bad taste and the appearance of oral ulcers significantly decreased in the experimental group (*p* <0.001). In addition, many of them had agreed that they could chew better and consequently had a better diet (*p* <0.001) after cancer therapy. We believe that this improvement in the patients’ quality of life is due to the regulated dental therapies they underwent and that led them to a considerable improvement in the quality of life.

The relevance of our study is that it allows us to assess the importance of dental treatment on the quality of life of patients with oral cancer among two study groups. There are no publications in the literature that observe the improvement in patients’ quality of life, thanks to regulated dental treatment, throughout cancer treatment (before, during and after).

Still, our study has some limitations. We only included patients undergoing chemoradiotherapy and excluded patients undergoing surgical treatment with tumor resection and those who were edentulous. The quality of life in patients undergoing surgical therapies can be highly committed at the nutritional, psychological, social and aesthetic levels, and although this has not been specifically evaluated in our study, our data could be extrapolated cautiously in these patients.

In conclusion, we believe that the implementation of prevention protocols and the improvement in oral health among these patients is necessary. Likewise, treatment of the oral pathology of cancer patients should be done at a multidisciplinary hospital level in a sphere where the dentist could have complete communication with the rest of the team involved in this pathology (nurses, nutritionists, psychologists, oncologists and oral and maxillofacial surgeons).
